# Epigenetic regulation of apoptosis and cell cycle regulatory genes in human colon carcinoma cells

**DOI:** 10.1016/j.gdata.2015.05.043

**Published:** 2015-06-12

**Authors:** Amy V. Paschall, Kebin Liu

**Affiliations:** Department of Biochemistry and Molecular Biology, and Cancer Center, Georgia Regents University, Augusta, GA 30912, USA

**Keywords:** Cell cycle arrest, Histone methyltransferase, Colon cancer, Apoptosis, H3K9 methylation

## Abstract

5-Fluorouracil (5-FU) is the standard chemotherapy for certain high risk stage 2 and all stage 3 and 4 human colorectal cancer patients. However, patients often develop chemoresistance to 5-FU. We have identified verticillin A from *Verticillium*-infected wild mushrooms as a potent anti-cancer agent that effectively suppresses 5-FU-resistant human colon cancer cells. Interestingly, a sublethal dose of verticillin A also acts as a potent sensitizer that overcomes human colon carcinoma cell resistance to FasL- and TRAIL-induced apoptosis. To identify verticillin A-regulated genes, we performed a genome-wide gene expression analysis and identified 1287 genes whose expression levels were either up- or down-regulated 1.5 fold. Forty-six of these genes have known function in regulation of apoptosis, and ninety genes have function in cell cycle regulation. Our recent study has identified verticillin A as a selective histone methyltransferase inhibitor. These identified genes are thus potential molecular targets for epigenetic-based therapy to overcome human colon cancer 5-FU resistance. The entire dataset is deposited in the NIH GEO database; accession number GSE51262.

**Table:** 

Specifications	
Organism/cell line/tissue	Cell line: Human colon carcinoma cell line LS411N-5FU-R. LS411N-5FU-R cell line was generated by culturing LS411N cells in the presence of 5-FU. LS411N cell line was established from Dukes' type B colon carcinoma tissue of a 37 year old patient. LS411N-5FU-R cells grow in the presence of 5-FU as high as 2.0 mg/ml in the culture medium.
Sex	Male colon cancer patient.
Sequencer or array type	Affymetrix Human Gene 2.0 ST Array
Data format	Raw and analyzed
Experimental factors	Cells were cultured in the absence (control) or presence of 50 nM verticillin A for 3 days. The gene expression level of treated cells was compared to the untreated cells.
Experimental features	
Consent	Exempted human colon cancer cell lines.
Sample source location	LS411N cell line was obtained from ATCC.

## 1 Direct link to deposited data

http://www.ncbi.nlm.nih.gov/geo/query/acc.cgi?acc=GSE51262.

## 2 Experimental design, materials and methods

The human colon carcinoma cell line LS411N was obtained from ATCC (Manassas, VA). Verticillin A was purified either from mushroom or fungus fermentation as previously described [1], [2]. To identify verticillin A-regulated genes, the 5-FU-resistant LS411N-5FU-R cells were cultured in the absence (control) or presence of verticillin A (50 nM) for three days. Total RNA was then isolated from cells using TRIzol according to the manufacturer's instructions (Life Technologies) as previously described [3], [4]. Biotinylated cDNAs were prepared according to the standard Ambion and Affymetrix protocol from 250 ng total RNA (The Ambion WT Expression Kit and GeneChip Terminal Labeling Kit, Affymetrix). Following labeling, cDNAs were hybridized for 16 h at 45 °C on Affymetrix Human Gene 2.0 ST Array. GeneChips were washed and stained in the Affymetrix Fluidics Station 450. GeneChips were scanned using the Affymetrix GeneChip Scanner 3000. Intensities of arrays have been quantile-normalized using Partek Genomic Suite (v6.6). Differential expressions were calculated using ANOVA of Partek package.

**Fig. 1:**
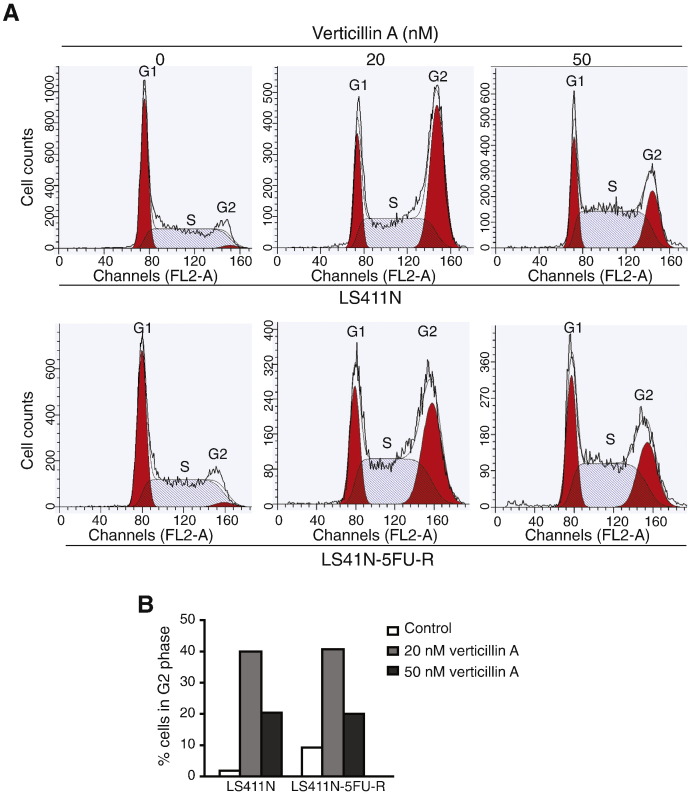
Verticillin A mediates cell-cycle progression in 5-FU-resistant human colon carcinoma cells. A. LS411N-5FU-R cells were cultured in the presence of Verticillin A at the indicated concentrations for 24 h. Cells were then stained with PI, and analyzed by flow cytometry. A. Cells as shown in A were quantified for percentages of cells in various phases the cell cycle (as shown). Columns, mean; bars, SD.

The entire data set was analyzed for differentially expressed genes. Using a 1.5 fold change and *p* value less than 0.01, we have identified 1287 genes whose expression levels were either up- or down-regulated 1.5 fold. Among these genes, forty-six have known function in regulation of apoptosis (Table 1), and ninety have function in cell cycle regulation (Table 2). Consistent with the altered gene expression in cell cycle regulation, functional analysis revealed that a sublethal dose of verticillin A induces cell cycle arrest at G2 phase (Fig. 1A and B). Comparison of effects of verticillin A on cell cycle arrest between the parent LS411N and the LS411N-5FU-R cells indicate that development of resistance to 5-FU does not alter human colon carcinoma cell sensitivity to verticillin A in induction of cell cycle arrest (Fig. 1B). Induction of cell cycle arrest is a mechanism by which chemotherapeutic agents suppress tumor development. Our observation that verticillin A induces cell cycle arrest in 5-FU-resistant human colon carcinoma cells as effectively as in the parent cells (Fig. 1B) suggests that verticillin A is potentially an effective agent for 5-FU-resistant cancer suppression [5], [6]. In summary, our data indicate that verticillin A-regulated genes, including these involved in apoptosis and cell cycle regulation, are potential molecular targets for epigenetic-based therapy [7], [8] to overcome human colon cancer 5-FU resistance.

## Figures and Tables

**Table 1:** Apoptosis regulatory genes.

Gene symbol	RefSeq	p value	Fold change
SORD	ENST00000267814	0.00061649	− 1.87556
ZMAT3	ENST00000311417	0.000557593	1.57282
ING3	ENST00000315870	0.000102115	− 1.51561
CLU	ENST00000316403	0.00658658	1.88366
ZADH2	ENST00000322342	0.00116724	− 1.45788
MALT1	ENST00000348428	8.70E− 05	− 1.59075
DNAJB4	ENST00000370763	0.000141036	1.57462
GADD45A	ENST00000370986	0.00036571	− 1.62913
SMOX	ENST00000379460	0.000453598	− 1.7213
FAS	NM_000043	0.00562506	1.64701
PMP22	NM_000304	0.0117398	1.80063
TP53	NM_000546	3.20E− 06	− 1.71073
OR51B5	NM_001005567	5.42E− 05	1.47933
MYBL1	NM_001080416	0.000313286	1.60353
CRYZ	NM_001130042	1.48E− 06	1.48424
JDP2	NM_001135049	0.00306543	− 1.60104
BIRC3	NM_001165	1.43E− 05	2.0818
EMP1	NM_001423	8.59E− 08	3.35724
EMP3	NM_001425	0.0010598	1.45705
CNTN1	NM_001843	0.0074873	− 1.56199
EDNRA	NM_001957	0.0279268	− 1.6314
FKBP4	NM_002014	9.38E− 05	1.46074
FYN	NM_002037	0.000281551	− 1.71704
LIF	NM_002309	0.0155574	− 1.45691
MAGEB2	NM_002364	0.00564587	1.57381
MDM2	NM_002392	4.74E− 05	1.96004
MYBL2	NM_002466	0.000287216	1.46171
ROBO1	NM_002941	0.000219031	− 1.87573
TXN	NM_003329	2.62E− 06	1.47208
CBX4	NM_003655	0.00057116	− 1.65897
BAG3	NM_004281	0.000615002	1.68888
CEBPB	NM_005194	3.37E− 05	− 1.55513
HDAC5	NM_005474	0.00951344	− 1.57188
DNAJB1	NM_006145	0.000604263	1.46166
FKBP9	NM_007270	0.000399435	− 1.49977
SHC2	NM_012435	0.015437	− 1.62921
CAPN6	NM_014289	0.000292268	− 1.62879
KRCC1	NM_016618	0.000625115	− 1.51118
SHC3	NM_016848	9.40E− 05	− 1.79462
FKBP10	NM_021939	0.00031242	− 1.59534
TMEM47	NM_031442	1.92E− 06	− 2.58264
IL20RB	NM_144717	3.47E− 06	− 2.65592
UNC5B	NM_170744	0.00192834	− 1.52166
IFNE	NM_176891	0.00165318	− 2.26639
RASSF3	NM_178169	0.000187772	− 1.47727
TYMS	NM_001071	0.000178443	1.46165

**Table 2:** Cell cycle regulatory genes.

Gene symbol	RefSeq	p value	Fold change
VRK1	ENST00000216639	0.00109304	1.48503
GINS2	ENST00000253462	0.000631431	1.55066
POLA2	ENST00000265465	0.000337543	1.50477
SKP2	ENST00000274255	0.000407268	1.4572
PLK2	ENST00000274289	0.000151913	1.72602
DUSP6	ENST00000279488	7.20E− 06	− 2.10422
ACTR1B	ENST00000289228	0.00108964	− 1.51729
FGF19	ENST00000294312	5.05E− 05	− 2.13694
CDC25A	ENST00000302506	0.000648382	1.56293
ESCO2	ENST00000305188	0.00119695	1.53802
PSMD2	ENST00000310118	0.000687665	1.49718
DSCC1	ENST00000313655	0.00380772	1.49838
ING3	ENST00000315870	0.000102115	− 1.51561
TUBB6	ENST00000317702	0.000718394	1.62504
NR2F1	ENST00000327111	0.000840833	− 1.73635
KLHDC1	ENST00000359332	0.00198403	− 1.85646
S100A10	ENST00000368811	0.000119039	1.45917
GADD45A	ENST00000370986	0.00036571	− 1.62913
JUN	ENST00000371222	4.77E− 05	− 1.92902
PLK3	ENST00000372201	0.000951583	1.49099
BICC1	ENST00000373886	0.000196311	− 1.5164
TTK	ENST00000509894	0.000571488	1.49117
PMP22	NM_000304	0.0117398	1.80063
TP53	NM_000546	3.20E− 06	− 1.71073
HNRNPA1L2	NM_001011724	0.0265379	− 1.55219
VEGFA	NM_001025366	7.18E− 06	− 2.08509
INCENP	NM_001040694	5.34E− 06	1.70379
MYBL1	NM_001080416	0.000313286	1.60353
PABPC1L	NM_001124756	0.000496661	− 1.98338
JDP2	NM_001135049	0.00306543	− 1.60104
FBXL17	NM_001163315	0.00683003	− 1.45608
ELAVL2	NM_001171197	0.0107444	− 1.83695
A1CF	NM_001198819	0.00310545	− 1.53498
CDKN1A	NM_001220778	0.0376178	1.47916
CCNE1	NM_001238	1.80E− 05	1.89998
DTNA	NM_001390	0.000846729	− 1.60955
EMP1	NM_001423	8.59E− 08	3.35724
EMP3	NM_001425	0.0010598	1.45705
CNTN1	NM_001843	0.0074873	− 1.56199
EGR1	NM_001964	4.11E− 05	− 2.05333
FKBP4	NM_002014	9.38E− 05	1.46074
MYBL2	NM_002466	0.000287216	1.46171
PCNA	NM_002592	7.01E− 06	1.80038
MAPK4	NM_002747	0.00147303	− 1.53275
ROBO1	NM_002941	0.000219031	− 1.87573
TXN	NM_003329	2.62E− 06	1.47208
CDKL2	NM_003948	0.0503581	1.57628
ORC1	NM_004153	1.65E− 05	1.93504
BUB1	NM_004336	4.67E− 07	1.70252
ETV5	NM_004454	4.20E− 05	− 1.65774
RPS6KA5	NM_004755	9.56E− 06	− 2.43124
ETV1	NM_004956	2.61E− 05	− 1.74844
E2F1	NM_005225	0.000685259	1.62065
JUND	NM_005354	0.000591427	− 1.61353
SKIL	NM_005414	6.76E− 05	− 1.71684
HDAC5	NM_005474	0.00951344	− 1.57188
UST	NM_005715	0.00693733	− 1.47903
KIF20A	NM_005733	0.000183352	1.66921
RASGRP1	NM_005739	0.00120733	− 1.49275
SFN	NM_006142	2.54E− 05	2.33324
POLH	NM_006502	2.21E− 05	2.1575
BTG2	NM_006763	4.54E− 05	1.54606
ZFP36L2	NM_006887	0.00908993	− 1.48935
RAPGEF4	NM_007023	0.010428	− 1.48297
POLI	NM_007195	0.00109012	− 1.57536
FKBP9	NM_007270	0.000399435	− 1.49977
HS2ST1	NM_012262	2.62E− 05	− 1.49078
ESPL1	NM_012291	0.00490513	1.47314
SESN1	NM_014454	0.0014303	1.5657
RPS6KA6	NM_014496	0.00411955	− 1.6027
HUNK	NM_014586	0.00831223	− 1.45803
MYO1D	NM_015194	0.00043732	− 1.62238
TUBE1	NM_016262	0.000107931	− 2.14386
FKBP10	NM_021939	0.00031242	− 1.59534
MOB3B	NM_024761	0.0171544	− 1.48453
DUSP16	NM_030640	0.00020683	− 1.46829
SESN2	NM_031459	2.47E− 05	− 2.27913
CCNB1	NM_031966	0.000656847	1.54547
GINS4	NM_032336	0.000779725	1.67209
BRSK1	NM_032430	0.00851809	− 1.63611
CORO6	NM_032854	0.000911746	1.49868
CDKN2B	NM_078487	6.95E− 05	− 2.14147
MSI2	NM_138962	0.00017894	− 1.47847
SLFN5	NM_144975	8.48E− 06	− 2.23313
DBF4B	NM_145663	0.000273346	1.55948
SIK1	NM_173354	0.00486314	1.49766
PTPDC1	NM_177995	2.21E− 05	− 2.35451
WDR49	NM_178824	0.0202432	1.5734
AURKA	NM_198433	0.00160995	1.62216
POLQ	NM_199420	3.09E− 05	1.62956

## References

[1] Liu F., Liu Q., Yang D., Bollag W.B., Robertson K., Wu P. (2011). Verticillin a overcomes apoptosis resistance in human colon carcinoma through DNA methylation-dependent upregulation of BNIP3. Cancer Res..

[2] Figueroa M., Graf T.N., Ayers S., Adcock A.F., Kroll D.J., Yang J. (2012). Cytotoxic epipolythiodioxopiperazine alkaloids from filamentous fungi of the Bionectriaceae. J. Antibiot..

[3] Paschall A.V., Zhang R., Qi C.F., Bardhan K., Peng L., Lu G. (2015). IFN regulatory factor 8 represses GM-CSF expression in T cells to affect myeloid cell lineage differentiation. J. Immunol..

[4] Paschall A.V., Zimmerman M.A., Torres C.M., Yang D., Chen M.R., Li X. (2014). Ceramide targets xIAP and cIAP1 to sensitize metastatic colon and breast cancer cells to apoptosis induction to suppress tumor progression. BMC Cancer.

[5] Klajic J., Busato F., Edvardsen H., Touleimat N., Fleischer T., Bukholm I. (2014). DNA methylation status of key cell-cycle regulators such as CDKNA2/p16 and CCNA1 correlates with treatment response to doxorubicin and 5-fluorouracil in locally advanced breast tumors. Clin. Cancer Res..

[6] Montagnoli A., Moll J., Colotta F. (2010). Targeting cell division cycle 7 kinase: a new approach for cancer therapy. Clin. Cancer Res..

[7] Greer E.L., Shi Y. (2012). Histone methylation: a dynamic mark in health, disease and inheritance. Nat. Rev. Genet..

[8] Crea F., Nobili S., Paolicchi E., Perrone G., Napoli C., Landini I. (2011). Epigenetics and chemoresistance in colorectal cancer: an opportunity for treatment tailoring and novel therapeutic strategies. Drug Resist. Updat..

